# The Role of Yeasts as Biocontrol Agents for Pathogenic Fungi on Postharvest Grapes: A Review

**DOI:** 10.3390/foods10071650

**Published:** 2021-07-16

**Authors:** Alessandra Di Canito, María Alejandra Mateo-Vargas, Monica Mazzieri, Jesús Cantoral, Roberto Foschino, Gustavo Cordero-Bueso, Ileana Vigentini

**Affiliations:** 1 Department of Food, Environmental and Nutritional Sciences, Università degli Studi di Milano, Via G. Celoria 2, 20133 Milan, Italy; alessandra.dicanito@unimi.it (A.D.C.); monicamazzieri7@gmail.com (M.M.); roberto.foschino@unimi.it (R.F.); 2 Department of Biomedicine, Biotechnology and Public Health, Universidad de Cádiz, Av. República Saharaui s/n, 11510 Cádiz, Spain; alejandra.mateovargas@alum.uca.es (M.A.M.-V.); jesusmanuel.cantoral@uca.es (J.C.)

**Keywords:** biocontrol, bioprotection, yeasts, non-*Saccharomyces*, *Aspergillus*, *Penicillium*, *Botrytis cinerea*, table grapes, vinification grapes, raisins, sustainability

## Abstract

In view of the growing concern about the impact of synthetic fungicides on human health and the environment, several government bodies have decided to ban them. As a result, a great number of studies have been carried out in recent decades with the aim of finding a biological alternative to inhibit the growth of fungal pathogens. In order to avoid the large losses of fruit and vegetables that these pathogens cause every year, the biological alternative’s efficacy should be the same as that of a chemical pesticide. In this review, the main studies discussed concern *Saccharomyces* and non-*Saccharomyces* yeasts as potential antagonists against phytopathogenic fungi of the genera *Penicillium* and *Aspergillus* and the species *Botrytis cinerea* on table grapes, wine grapes, and raisins.

## 1. Introduction

Among microorganisms with food and industrial interest, yeasts are one of the most significant/important agents. This is especially true for *Saccharomyces cerevisiae*, which has been used for centuries in wine, beer, and bread production and which was the first genetically manipulated eukaryote [1]. In addition, the so-called non-conventional yeast species—e.g., *Kluyveromyces lactis* (Dombrowski) van der Walt (1971)), *Debaryomyces hansenii* ((Zopf) Lodder & Kreger-van Rij), *Zygosaccharomyces rouxii* ((Boutroux) Yarrow), and *Z. bailii* ((Lindner) Guilliermond (1912))—play a role in traditional food processes [2]. However, several non-conventional species remain largely unexplored both in basic research and for their possible commercialization. This constitutes a huge, untapped reservoir of potential biotechnological innovations which involve the selection of species and strains with new metabolic traits such as the secretion of proteins, adhesiveness, antimicrobial properties, etc. Several genomes of non-conventional yeast species have been completely sequenced and their number continues to grow [3]. Thus, novel methods for the genetic analysis and modification of yeasts, as well as their genomic and post-genomic analysis, will represent a platform for understanding the molecular mechanisms underlying both the simple and complex biological features that are useful for the development of novel and eco-compatible applications.

Nowadays, the vitiviniculture sector is highly motivated to develop sustainable approaches to counteract climate change, which affects the entire wine industry across the world. In particular, some areas are affected by increasingly widespread rainfall with the risk of hydrogeological instability, whereas others are affected by unprecedented droughts and heat waves.

From a microbiological point of view, climate change could increase the risk of plant diseases due to the proliferation of pathogenic fungi belonging to the genera *Botrytis*, *Penicillium*, and *Aspergillus*. Their uncontrolled proliferation could cause huge economic losses. This situation has often been managed with the use of pesticides, whose abuse led the European community to establish rules for the sustainable use of agrichemicals to reduce risks and impacts on both people’s health and the environment through the EU Directive 2009/128/CE. Moreover, this directive promotes the use of integrated defense and different approaches or techniques, such as *non*-chemical alternatives to pesticides, including a biological control approach.

The term “biological control” was used for the first time by Smith (1919) to describe the introduction of natural enemies of exotic insects for the permanent suppression of insect pests [4]. In general, this term includes practically all pest control measures except the application of chemicals. In particular, the use of microorganisms selected as biocontrol agents (BCAs) was later introduced by Baker and Cook (1974) [5]. They defined biocontrol as the reduction in pathogen presence or disease activities by the introduction of one or more antagonist organisms. Biocontrol is successfully applied for constitutive or induced resistance that can be triggered by natural plant products and/or antagonistic microorganisms to control pathogens [6,7]. According to the definition of biocontrol in the agri-food sector, this approach refers to a set of emerging strategies that are alternatives to the use of chemicals for combatting fruit and vegetable diseases. Moreover, biocontrol also extends to food production and preservation [8,9]. Innovative strategies in biocontrol include the use of selected microorganisms or BCAs, with antagonistic activity against other microorganisms, reducing the use of pesticides [6] and boosting food quality and safety [10,11].

**Figure 1:**
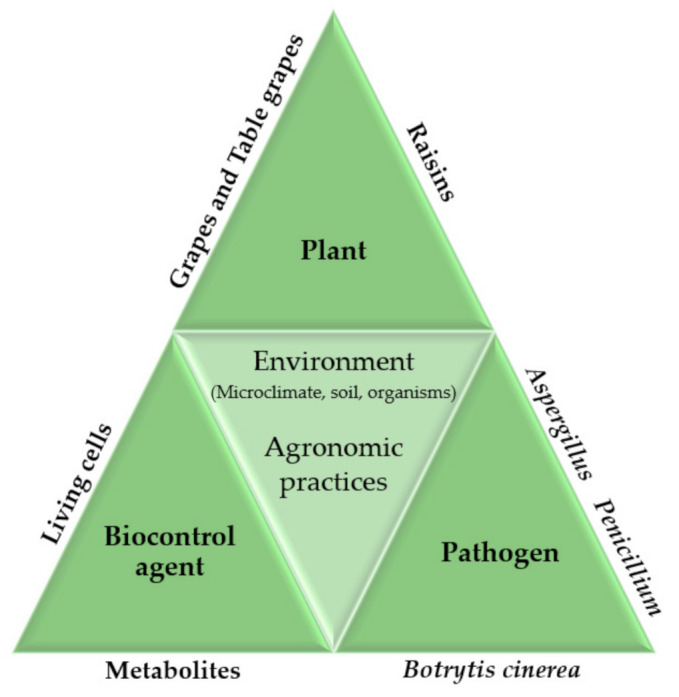
Evolution of biocontrol research towards the integration of microbial communities into the current research triangle (re-adapted by [18]).

The criteria for the selection of an ideal BCA are the following: It must be genetically stable, effective at a low concentration, not fastidious in its nutritional requirements, capable of surviving under adverse environmental conditions, effective against a wide range of pathogens and different harvested commodities, resistant to pesticides, a non-producer of metabolites harmful to humans, non-pathogenic to the host, in a storable form, and compatible with other chemical and physical treatments. In addition, a microbial antagonist should have an adaptive advantage over specific pathogens [6,7]. Recently, research has aimed at improving their performance in order for them to be used as biopesticides, starting from a thorough examination of the physiological and molecular mechanisms of interaction among all the parties involved (plant, pathogen/parasite, and BCA) to increase the breadth of the BCA spectrum of action [12]. In 1988, Pusey et al. tested and subsequently patented a *Bacillus subtilis* ((Ehrenberg) Cohn) strain in the USA as the first biocontrol microorganism in post-harvest peach brown rot disease in combination with 2,6-dichloro-4-nitroaniline, water-based wax, paraffin, and mineral oil base [13]. Then, in 1991, the same researchers successfully applied and patented *B. subtilis* on post-harvest apples and grapes to inhibit the growth of brown, gray, and bitter rots [14]. Later, the yeast species *Rhodotorula glutinis* ((Fresenius) Harrison) and *Rhodotorula mucilaginosa* ((Jorgensen) Harrison) were patented as biocontrol agents against mold, the causative agents of gray and blue molds, along with mucor and transit rots of fruit [15]. Subsequently, Wilson et al. in 1998 released a patent on *Candida oleophila* (Montrocher (1967)) as an agent against post-harvest diseases caused by *Penicillium expansum* ((Persoon) Saccardo), *Penicillium digitatum* ((Persoon) Saccardo), and *Botrytis cinerea* (Persoon (1794)) [16]. Meanwhile, in 2006, the yeast *Mestchnikowia fructicola* was used against the pathogenic fungi *B. cinerea*, *P. digitatum*, and *Aspergillus niger* (van Tieghem), being able to reduce the post-harvest decay of fruit through competitive inhibition [17]. The advent of high-throughput sequencing (or next-generation sequencing, NGS) technologies is now driving a paradigm change, allowing researchers to integrate microbial community studies into the traditional biocontrol approach [18] (Figure 1).

Alternatively, it could be interesting to investigate the use of endophytic microorganisms as BCAs. Indeed, fungi and bacteria living in plant tissues can have beneficial effects without causing disease [19,20], such as providing protection against pathogens and environmental stressors [21,22,23]. Several studies have revealed that fungal endophytes produce a variety of effects in their host, such as the release of phytohormones [24] and/or molecules useful for preventing certain plant diseases [25,26,27]. Considering that endophytes occupy the same niche as phytopathogens in plants and compete for space and nutrients, harnessing them as BCAs could represent an innovative way to counteract plant diseases and reduce the utilization of pesticides. In particular, evidence regarding the presence of endophytic grape yeasts belonging to the genera *Metschnikowia*, *Pichia*, and *Hanseniaspora* has been reported in the literature [28,29]. Considering ideal expression systems [30], endophytic yeasts will gain increasing interest for future biotechnological applications as biocontrol agents in plants.

In a period of almost 60 years, from 1963 to 2021, numerous studies have been conducted on the application of biocontrol mechanisms in the agri-food sector. Since 1990, several studies on biocontrol agents of grape pathogens have been carried out. Although some studies have been carried out on the biocontrol of pre- and post-harvest grape, about 48% of the available literature concerns investigations of postharvest grape. Thus, considering the growing interest in the potential role of yeasts as BCAs [30] and the relatively fragmented information related to single species and strains, this review aims to summarize the current knowledge regarding yeast activity against pathogenic fungi on postharvest grapes. Moreover, several pathogens and consequently new yeast species with the potential to be used as BCAs have recently been discovered and citations about their roles are reported here. However, this review was inspired by the scarcity of information related to the biocontrol activity of yeasts against *Aspergillus* spp., *Penicillium* spp., and *Botrytis cinerea* species—i.e., the three main causes of postharvest grape diseases [31,32,33,34]. Finally, the present work analyzes the relevant mechanisms underlying the antagonistic action of yeasts on the above-mentioned molds and presents a comprehensive table with reference material for a rapid and direct comparison.

## 2. BCA Mechanisms of Action

At present, the utilization of BCAs is growing quickly in the wine sector since research is shedding light on how they behave (their *mode of action*), which is key to determining their potential success in industrial applications.

One of the most studied biocontrol mechanisms of action of BCAs is the competition for space and nutrients. This mechanism assumes great importance in post-harvest treatments since numerous infections originate from wounds caused during the collection, selection, packaging, and marketing phases of grapes. Competition is an effective biocontrol activity if the antagonist is present at the same time and location in the pathogen in a sufficient amount, limiting the resources available [34]. Another relevant approach is iron competition, as this involves the antagonist production of siderophores, small molecules with a high affinity for iron and that are capable of chelating Fe^3+^ ions. This is important in the biocontrol exerted by molds, since iron is fundamental for their growth and for the pathogenesis process [34]. Furthermore, biofilm formation and resistance induction are other possible mechanisms. The formation of biofilms is a specific strategy for the space competition used by BCAs to successfully colonize intact or damaged fruit surfaces and better promote adherence and multiplication.

The induction of resistance in plants consists of stimulating the activation of defense mechanisms by means of elicitors, which are synthetic or natural molecules that mimic the attack of a pathogen or a state of stress. This mechanism can involve the activation of pathogenesis-related proteins (β-1,3-glucanases and chitinases) or defense-related enzymes, such as phenylalanine ammonia-lyase (PAL), peroxidase, and polyphenoloxidase, which are produced by biocontrol agents [35,36].

The production of primary and secondary metabolites, particularly against filamentous fungi, is considered a crucial mechanism of action of BCAs. Among metabolites, volatile organic compounds (VOCs) have a high biocontrol efficacy. They are small (usually <300 Da) molecules with a low solubility in water and a high vapor pressure. VOCs include several molecular classes that show a high spectrum of action as antimicrobials, such as hydrocarbons, alcohols, thioalcohols, aldehydes, ketones, thioesters, cyclohexanes, heterocyclic compounds, phenols, and benzene derivatives [34,35]. This mechanism has great efficacy in the in vitro tests, but when carrying out trials on the fruit surface the applied VOC concentration must be carefully finetuned. Killer toxins are also efficient metabolites for biocontrol. They are proteins or glycoproteins that are lethal for pathogenic fungi or secondary metabolites that are able to inhibit the proliferation of molds on table grapes and grapes [34]. Additionally, the secretion of lytic enzymes by BCAs, such as glucanases, chitinases, lipases, and proteases, is a common feature in all types of host–pathogen interactions and has been extensively studied [34,35].

## 3. Yeast Applications against Pathogenic Fungi of the Grape in Post-Harvest

From an economic and social point of view, the grape (*Vitis vinifera* L.) is one of the most widely cultivated fruit plants in the world and is susceptible to infections caused by pathogenic fungi [36]. Therefore, in recent times, the reduction in phytopathogenic fungi attacking the grape has also become of vital interest for vine growers in the post-harvest phase [37]. Microbiological applications to prevent infections represent a new strategic frontier for maintaining the post-harvest quality of table and wine grapes [38,39]. A study conducted in 2000 stated that the application of BCAs in the field allows the early colonization of fruit surfaces, thus protecting against latent infections that occur after harvest. In particular, over the past 20 years the role and mechanisms of yeast activity as a biocontrol against pathogenic fungi have been assessed and described (Table A1, Appendix A).

The pathogenic fungi primarily studied are *Aspergillus* spp., *Penicillium* spp., and *B. cinerea*. However, other grape pathogenic fungi have lately been mentioned in the literature, such as *Rhizopus stolonifera* ((Ehrenberg) Vuillemin), *Mucor piriformis* (A. Fischer), *Colletotrichum acutatum* (J.H. Simmonds), *Alternaria* sp. (Nees), and *Rhizoctonia* sp. (Kühn) [40,41]. The yeasts which exert biocontrol activity against these fungi are *Trichoderma harzianum* (Rifai), *Pichia membranifaciens* (E.C. Hansen (1904)), *Kloeckera apiculata* ((Reess) Janke), *Candida guilliermondii* (Castellani) Langeron & Guerra var. carpophila Phaff & M.W. Miller (1961)), *Cryptococcus laurentii* ((Kufferath) CE Skinner), *Cryptococcus flavus*, *Cryptococcus albicus*, *Candida pyralidae* (Kurtzman (2001b)), *Pichia kluyveri* (Bedford ex Kudryavtsev (1960)), and *P. expansum* [40,41,42,43,44,45].

### 3.1. Biological Control against Aspergillus Genus

In recent years, several yeast species have been investigated and used as potential BCAs against *Aspergillus carbonarius* ((Bainier) Thom), which affect not only grapes but also other fruits. Fungi belonging to the *Aspergillus* genus are generally responsible for the release of mycotoxins, which are compounds that are harmful to humans, including ochratoxin A [46,47,48], for which extensive legislation has been passed (Regulation (EC) no. 401/2006). Notably, it was demonstrated that the yeasts *Trichosporon mycotoxinivorans* and *Yarrowia lipolytica* ((Wickerham, Kurtzman & Herman) van der Walt & von Arx) are able to degrade these toxins [37]. Moreover, in 2017, Cordero-Bueso et al. [49] selected four non-*Saccharomyces* yeasts (*P. kluyveri*, *Hanseniaspora uvarum* ((Niehaus) Shehata, Mrak & Phaff ex M.Th. Smith), *Meyerozyma (Pichia) guilliermondii* ((Wickerham) Kurtzman et M. Suzuki), and *Hanseniaspora clermontiae* (Cadez, Poot, Raspor & M.Th. Smith (2003))) to counteract the *A. carbonarius* infection of grape, and different mechanisms of action were reported. The study revealed that yeasts could inhibit the growth of *A. carbonarius* through competition for the substrate but not by iron competition. Furthermore, *P. kluyveri* and *H. uvarum* also acted through biofilm formation, the secretion of lytic enzymes, the induction of resistance, and the production of killer toxins and VOCs. On the other hand, the yeasts *M. guilliermondii* and *H. clermontiae* carried out biocontrol activity through the production of unidentified VOCs and enzymatic activity, respectively. However, regarding the production of VOCs, it is known that *Cyberlindnera jadinii* ((Sartory et al.) Minter), *Lanchea thermotolerans*, *Candida intermedia* ((Ciferri & Ashford) Langeron & Guerra (1938)), and *Candida friedrichii* (Uden & Windisch (1968)) can inhibit the growth of *A. carbonarius* and *Aspergillus ochraceus* through the production of 2-phenylethanol [50,51]. The proliferation of *A. niger* can be counteracted using the yeast *D. hansenii*, which works through the production of killer toxins [46]. *Saccharomyces cerevisiae* (Meyen ex E.C. Hansen (1883)), *Wickerhamomyces anomalus* ((Hansen) Kurtzman, Robnett & Basehoar-Powers (2008)), *Rhodosporidium fluviale,* and *Rhodosporidium paludigenum* were used to prevent the growth of *Aspergillus japonicas* (Saito), *Aspergillus uvarum*, and *Aspergillus aculeatus* (Lizuka) on table grapes post-harvest. These yeasts exhibited biocontrol activity through the production of lytic enzymes, with the exception of *W. anomalus*, which also produced killer toxins [52]. Recently, yeast species belonging to the genera *Saccharomyces*, *Pichia*, *Metschnikowia*, *Dekkera* (van der Walt), and *Rhodotorula* (Harrison (1928)) were found to be able to reduce the growth rate of *A. carbonarius*, thus offering a set of species potentially acting as antagonists of the pathogenic fungi involved in grape and wine production [53,54].

### 3.2. Biological Control against Penicillium Genus

*Penicillium* is one of the most common fungal genera in nature and its several species can proliferate in different habitats. Moreover, some of them are able to produce mycotoxins [55]. *P. expansum* produces both patulin (PAT) and citrinin (CIT), harmful compounds which provoke potentially high health risks [56]. Thus, the selection of BCAs against some detrimental species of *Penicillium* in the grape post-harvest phases is advised. Several non-*Saccharomyces* species show activity towards *P. expansum* and *P. digitatum* [49]. In particular, the production of VOCs by *Candida sake* ((Saito & Oda) van Uden & H.R. Buckley ex S.A. Meyer & Ahearn (1983)) yeast against the former appears effective [57]. *W. anomalus*, for example, counteracts the proliferation of *P. digitatum* through the release of killer toxins and lytic enzymes [58]. Additionally, *W. anomalus* is able to inhibit the growth of the pathogenic fungi *P. expansum, Starmerella bacillaris* (formerly *Candida zemplinina,* on apple in post-harvest) (Sipiczki (2003)), and *R. paludigenum* (on pear in post-harvest) by competition for the substrate, the induction of resistance, and the secretion of lytic enzymes (such as polyphenol oxidase, catalase, and chitinase) [59,60]. Additionally, *M. fructicola* was proven to possess an antagonistic activity based on iron competition, the induction of resistance, and the production of lytic enzymes against *P. digitatum* [61]. Recently, two species of *Penicillium* (Link (1809)), *P. rubens* (Biourge) [36], and *P. georgiense* (S.W.Peterson & B.W.Horn) [52], were isolated for the first time on table grapes and identified as post-harvest grape deterioration agents. *Y. lipolytica* counters *P. rubens* through the production of lytic enzymes [36]. The same mechanism of action is used by *S. cerevisiae*, *W. anomalus*, *R. fluviale*, and *R. paludigenum* in counteracting *P. georgiense* and *P. expansum*. Moreover, the inhibitory activity of *R. paludigenum* is also achieved by the induction of resistance and the production of killer toxins [52]. Finally, Yang et al. [62] tested the coupled use of vitamin C and *Pichia caribbica* (Vaughan-Martini, Kurtzman, S.A. Meyer & O’Neill (2005)) against *P. expansum*. The results showed that the application of vitamin C could improve the biological control against the fungus by increasing the metabolic activity of the yeast and promoting competition with the pathogen [63].

### 3.3. Biological Control against B. cinerea

The species *B. cinerea* (Persoon) is particularly relevant in the wine field for two fundamentally different reasons: It induces diseases in grapes and is also used in raisining wine technology. Since the research on the biocontrol activity of this species is very extensive, this paragraph provides a more thorough analysis than the previous.

*B. cinerea* is an airborne filamentous fungus that causes grey mold disease [64]. This necrotrophic phytopathogenic fungus is extremely polyphagous and ubiquitous, thus its action extends to almost all regions of the world with great losses in fruit and vegetable crops, including the grapevine [65]. Moreover, *B. cinerea* produces a polyketide mycotoxin, botcinic acid, during infection [66]. The growth of *B. cinerea* is commonly controlled through a combination of a fungicide treatment and specific agronomic practices. Indeed, although the latter approach helps minimize infections, it is not sufficient to prevent the disease caused by *B. cinerea* in many wine-growing areas [67].

Mechanisms of yeast action against the proliferation of *B. cinerea* have been reported to be mainly linked to the production of enzymatic activities, iron or nutrient competition, and the production of VOCs.

BCAs exerting biocontrol through enzyme production were first reported by Lima et al. in 1998 [68], when two strains belonging to *C. laurentii* (LS-28) and *R. glutinis* (LS-11) species were isolated on Italian table grapes. These yeasts showed a high biocontrol activity towards *B. cinerea* thanks to the secretion of β-1,3-glucanase. Later, other yeasts of the species *C. oleophila*, *D. hansenii*, *M. guilliermondii*, and *Metschnikowia* were tested in the in vivo tests on table grapes. In particular, the *Metschnikowia*-like yeast strain LS-15 significantly reduced the grey fungus (from 28.3% to 38.2%) [69], such as *M. fructicola* (strain NRRL Y-27328, CBS 8853), which showed its possible capability to inhibit *Botrytis* rot in stored grapes [70]. Additionally, *P. membranifaciens* FY-101 exhibited antagonistic properties against *B. cinerea*, preventing the symptoms of grey rot disease in *V. vinifera* and *Vitis rupestris*, probably due to the secretion of β-1,3-glucanases [71,72]. Moreover, in 2015, Parafati et al. [73] analyzed the effectiveness of *W. anomalus*, *M. pulcherrima*, and *Aureobasidium pullulans*, isolated from different food sources, as BCAs against *B. cinerea*. *W. anomalus* strains were selected for their high kill capacity against the sensitive *S. cerevisiae* strain, identifying β-glucanase as responsible for the toxicity mechanism. In vitro studies revealed that they were able to reduce mycelial growth with a variable efficacy. Furthermore, while *M. pulcherrima* and *W. anomalus* strains showed greater inhibition at pH 4.5 (72.67% and 81.50%, respectively), the *A. pullulans* strains maintained the same activity at different pH values (such as 70.89% and 71.33%, respectively, at pH 6 and 4.5). Regarding the enzymatic activities, only *A. pullulans* and *W. anomalus* strains showed a β-1,3-glucanase activity capable of hydrolyzing laminarin. Additionally, the *A. pullulans* strains showed pectinolytic and proteolytic activity.

The iron competition mechanism against *B. cinerea* was elucidated in *M. pulcherrima* (MACH1) [74]. In culturing MACH1 with different iron concentrations (supplemented as FeCl_3_) together with *B. cinerea*, it was observed that the yeast strain produced a wide pigmented zone of inhibition when the iron concentrations were low. In addition, in the inhibition zones, the conidia of the pathogen did not germinate and mycelial degeneration was observed. Furthermore, in vivo experiments on apples supplemented with low iron conditioning revealed a greater reduction in infection compared to a high iron conditioning state. Thus, iron deficiency appears to be an important factor in the biocontrol exerted by yeast against various fungal pathogens. Indeed, as described in Parafati (2015) [73], *M. pulcherrima* strains synthesize pigments such as pulcherrimin, producing the widest zones of inhibition in absence of FeCl_3_ [75]. Later, Cordero-Bueso et al. [49] corroborated this mechanism of action against *B. cinerea* on grapes.

Competition for nutrients plays an important role in the *K. apiculata*’s biological control capability against *B. cinerea*. Specifically, the strain 34-9 isolated from citrus roots exhibited rapid colonization of grape wounds and a great ability to control *B. cinerea* in the in vivo and in vitro tests on grapes (*V. vinifera* L. × *Vitis labrusca* L. cv. Kyoho) [76]. In addition, *H. uvarum* (anamorph *K. apiculata*) was used in combination with tea polyphenols (TP) against *B. cinerea* on *V. vinifera* L. Kyoho. The results showed that TP significantly increased the yeast population and efficacy at the different tested concentrations. This case is an example of how the biocontrol activity exhibited by some antagonists can be improved [77].

In general, the composition of the microbiota appears to be relevant in the control of *B. cinerea,* as reported in a study carried out on grapes involved in the production of the Italian straw wine “Vino Santo Trentino”, which uses the “Nosiola” grape [78], where the *Hanseniaspora*, *Metschnikowia*, *Cryptococcus*, and *Issatchenkia* genera were identified. Their presence was intricately related to the extent of infection caused by *B. cinerea*. Moreover, these microorganisms are poorly resistant to ethanol and have low pH values, thus they did not represent a risk for either the winemaking process or the development of bad flavors, since they disappear during the process [78]. Another noticeable microbiota is the one identified on mummified grapes infected with *B. cinerea* during winter (noble rot) in the Tokaj region (Hungary) [79]. The presence of viable strains showed that mummified grapes could serve as a safe yeast reservoir and could contribute to maintaining these colonizing populations of grapes in the vineyard over time. Indeed, among the most frequent isolates it was possible to detect three species of *Hanseniaspora*, pigmented strains of *Metschnikowia*, three species of *Saccharomyces* (*S. paradoxus*, *S. cerevisiae*, and *S. uvarum*), *Aureobasidium subglaciale*, *Kabatiella microsticta* (Bubk (1907)), *Columnosphaeria fagi*, and W. *anomalus*. These yeasts maintained complex interactions with *B. cinerea*, including antagonism (growth and contact inhibition, competition for nutrients) and synergism (cross-feeding). The *Metschnikowia* strains showed antagonistic activity against *Botrytis*, inhibiting the germination of their conidia and the extension of their hyphae, probably due to competition for iron, while *S. paradoxus* and *S. uvarum* activities were associated with competition for nutrients [79].

Wang et al. (2018) [80] evaluated the biocontrol exerted by 10 non-*Saccharomyces* yeasts strains, isolated from vineyards, against *B. cinerea* on “Thompson seedless” table grapes. The isolates belonged to the species *A. pullulans*, *Candida saitoana* (Nakase & M. Suzuki (1985)), *Curvibasisium pallidicorallinum*, *Metschnikowia chrysoperlae*, *M. pulcherrima*, *M. guilliermondii*, and *W. anomalus*. All of them rapidly colonized grape berries, with *W. anomalus* being the most effective. The results suggested that the latter was the most effective and that the biocontrol mechanism of these yeasts was the competition for the niche.

Cordero-Bueso et al. (2017) investigated the potential biocontrol action of epiphytic non-*Saccharomyces* yeast strains isolated from grape berries from *V. vinifera* spp. *sylvestris* and *V. vinifera* spp. against *B. cinerea* [49]. Of the 19 antagonist strains belonging to seven different species, the most effective in reducing the incidence of *B. cinerea* were *H. clermontiae* (Cadez, Poot, Raspor & M.Th. Smith (2003)), *H. uvarum*, *M. guilliermondii*, and *P. kluyveri*. In particular, *P. kluyveri* strain SEHMA6B has shown to be more effective against *B. cinerea* than the synthetic fungicides. Moreover, in 2019, Carmichael et al. [81] evaluated the abundance and the diversity of yeast populations already known as natural antagonists of postharvest pathogens (*Aureobasidium*, *Cryptococcus*, *Rhodotorula*, and *Sporobolomyces*), with a particular focus on their action against *B. cinerea*, in the different phenological stages of table grapes (variety crimson seedless). The results of this study showed that a high presence of populations of possible biocontrol yeasts was associated with a low prevalence of pathogenic groups. The development stages of table grapes with low concentrations of *B. cinerea* presented an abundance of *Rhodotorula*, *Aureobasidium*, and *Cryptococcus*.

While the biocontrol mechanisms on table grapes have been widely investigated, the antagonism of yeasts against *B. cinerea* in raisin grapes is poorly documented. The work of Ribereau-Gayon in 1970 [82] suggested that *B. cinerea* acts as an active component in the raisins of certain grapes. The role of *Botrytis* depends on the environmental conditions. When the berry tissue remains intact until its maturity, *B. cinerea* causes a “noble putrefaction” that improves the constitution of the berry. It dries out the berry and increases the sugar concentrations but the acidity only slightly. If, on the contrary, there are injuries caused by insects, birds, worms or attacks by other fungi, *Botrytis* develops rapidly in this environment as a favorable pathogen. In this way, the concentration of sugars and acidity are strongly reduced.

Regarding the release of VOCs, Lemos et al. (2016) [83] isolated *S. bacillaris* species from the fermenting musts of overripe dry grapes (variety *Raboso Piave*). This variety was selected since the aging of the skin makes them more susceptible to infections caused by fungal pathogens, such as *B. cinerea*. In vitro tests indicated that the VOC production was primarily responsible for the antifungal effects exhibited by *S. bacillaris*. Then, in 2018, Kasfi et al. [84] isolated epiphytic microorganisms from the variety “Thompson seedless” in order to identify those that exerted biocontrol on *B. cinerea*. Among all the isolates, five yeast strains showed in vitro the best activity against *Botrytis*: Three were *M. guilliermondii* and two were *C. membranaefaciens*. Some of them inhibited the mycelial growth of the pathogen through VOCs. In 2017, Cordero-Bueso et al. showed a similar phenomenon in an *A. pullulans* yeast strain [49].

## 4. Conclusions

Pathogenic strains belonging to the genera *Aspergillus*, *Penicillium*, and *Botrytis* are perhaps the most challenging problem for fruit and vegetable crops around the world. These phytopathogenic fungi cause great economic losses every year and are a potential hazard for humans, so their control is essential in both pre-harvest and post-harvest grapes [35,85]. Regarding attempts to obtain an organic alternative to replace chemical pesticides, many studies have been carried out on yeast BCAs in the last few decades. If the efficacy of yeasts is confirmed, further studies will be required to assess the potential risk to human health.

Although epiphytic strains belonging to the genus *Saccharomyces* tend to be recurrent antagonists of these fungi, their efficacy as BCAs is frequently inferior to that of other non-*Saccharomyces* yeasts and synthetic fungicides. The non-*Saccharomyces* genera constitute a great variety of species that have shown effective antagonistic capacity against pathogenic fungi on grapes. Some studies have shown the possibility of further enhancing its action by combining it with other substances or organisms. Moreover, endophytic yeasts could soon become a resource in terms of BCAs. In fact, the genera *Metschnikowia*, *Pichia*, and *Hanseniaspora* have recently been detected in grapes [28,86].

The action of pathogenic mold antagonists in the in vitro experiments on postharvest grapes differs from the action that they exhibit in vivo. This highlights the importance of carrying out experiments under field conditions before marketing a product, as well as the suitability of evaluating different strains on grape berries to screen and determine their potential toxicity or find which ones are most effective against a pathogen. The difficulty in developing a commercial formulation is also accentuated by the many parameters that must be considered.

Regarding the type of grape used, the number of studies assessing the performance of BCAs on table grapes is considerably higher than the number of studies assessing their performance on wine grapes and raisins. In some of these, the microbiota of wine grapes was isolated to evaluate the activity of these possible antagonists on table grapes. Therefore, if the results were positive, it could be deduced that these BCAs would also demonstrate activity in wine grapes, although in vivo studies would be necessary to confirm this. On the other hand, the action of *B. cinerea* is not always negative, since under certain circumstances it can cause “noble putrefaction” on raisins, increasing the concentration of sugars and allowing the production of sweet wines such as “Vino Santo Trentino” or the Tokaj wines. Finally, since the interplay between pathogen, antagonist, and environment is highly complex, understanding the mechanisms and conditions at the root of the BCA action will enhance the effectiveness of future decisions made regarding biocontrol strategies.

## Appendix A

**Table A1:** Description of the mechanisms of action of potential biological control agents. ND: Not done; +: Biocontrol activity; -: Not biocontrol activity.

Genus	Species	Control Agents	Mechanism of Action								References
			Living Cells				Metabolite				
			Competition for Substrate	Competition for Iron	Killer Factor	Biofilm	VOCs	BCAs	Resistance Induction	Enzymatic Activity	
*Aspergillus*	*A. carbonarius*	*P. kluyveri*	+	-	-	+	-	ND	-	+	[49]
		*H. uvarum*	+	-	-	-	-	ND	-	-	[49]
		*M. guilliermondii*	+	-	-	-	+	ND	-	+	[49]
		*H. clermontiae*	+	-	-	-	-	ND	-	+	[49]
		*Cyberlindnera jadinii*	ND	ND	ND	ND	+	ND	ND	ND	[50]
		*C. friedrichii*	ND	ND	ND	ND	+	ND	ND	ND	[50]
		*C. intermedia*	ND	ND	ND	ND	+	ND	ND	ND	[50,51]
		*L. thermotolerans*	ND	ND	ND	ND	+	ND	ND	ND	[50]
		*S. cerevisiae*	+	ND	ND	ND	ND	ND	ND	+	[53,54]
	*A. niger*	*D. hansenii*	ND	ND	+	ND	ND	ND	ND	ND	[46]
	*A. ochraceus*	*Cyberlindnera jadinii*	ND	ND	ND	ND	+	ND	ND	ND	[50]
		*C. friedrichii*	ND	ND	ND	ND	+	ND	ND	ND	[50]
		*C. intermedia*	ND	ND	ND	ND	+	ND	ND	ND	[50]
		*L. thermotolerans*	ND	ND	ND	ND	+	ND	ND	ND	[50]
	*A. japonicas*	*S. cerevisiae*	ND	ND	ND	ND	ND	ND	ND	+	[52]
		*W. anomalus*	ND	ND	+	ND	ND	ND	ND	+	[52]
		*R. fluviale*	ND	ND	ND	ND	ND	ND	ND	+	[52]
		*R. paludigenum*	ND	ND	ND	ND	ND	ND	ND	+	[52]
	*A. uvarum*	*S. cerevisae*	ND	ND	ND	ND	ND	ND	ND	+	[52]
		*W. anomalus*	ND	ND	+	ND	ND	ND	ND	+	[52]
		*R. fluviale*	ND	ND	ND	ND	ND	ND	ND	+	[52]
		*R. paludigenum*	ND	ND	ND	ND	ND	ND	ND	+	[52]
	*A. aculeatus*	*S. cerevisiae*	ND	ND	ND	ND	ND	ND	ND	+	[52]
		*W. anomalus*	ND	ND	+	ND	ND	ND	ND	+	[52]
		*R.fluviale*	ND	ND	ND	ND	ND	ND	ND	+	[52]
		*R. paludigenum*	ND	ND	ND	ND	ND	ND	ND	+	[52]
*Penicillium*	*P. expansum*	*P. kluyveri*	+	+	-	+	-	ND	-	+	[49]
		*H. uvarum*	+	-	-	-	-	ND	-	-	[49]
		*M. guilliermondii*	+	-	-	-	+	ND	-	+	[49]
		*H. clermontiae*	+	-	-	-	-	ND	-	+	[49]
		*Candida sake*	-	ND	ND	+	ND	ND	ND	ND	[57]
		*Starmerella BCAillaris*	+	ND	ND	ND	ND	ND	ND	ND	[60]
		*W. anomalus*	ND	ND	+	ND	ND	ND	+	+	[49,52]
		*S.cerevisiae*	ND	ND	ND	ND	ND	ND	ND	+	[52]
		*R.fluviale*	ND	ND	ND	ND	ND	ND	ND	+	[52]
		*R. paludigenum*	ND	ND	ND	ND	ND	ND	+	+	[52,61]
		*P. carribica + Vitamin C*	ND	ND	ND	ND	ND	ND	+	+	[51]
	*P. digitatum*	*W. anomalus*	ND	ND	+	ND	ND	ND	ND	+	[62]
		*M. fructicola*	ND	+	ND	ND	ND	ND	+	+	[50]
	*P. georgiense*	*S. cerevisiae*	ND	ND	ND	ND	ND	ND	ND	+	[52]
		*W. anomalus*	ND	ND	+	ND	ND	ND	ND	+	[52]
		*R. fluviale*	ND	ND	ND	ND	ND	ND	ND	+	[52]
		*R. paludigenum*	ND	ND	ND	ND	ND	ND	+	+	[52]
	*P. rubens*	*Y. lipolytica*	+	ND	ND	ND	ND	ND	ND	+	[49]
